# Within-subject confidence intervals for pairwise differences in scatter plots

**DOI:** 10.3758/s13423-025-02750-1

**Published:** 2025-08-28

**Authors:** Alexander C. Schütz, Karl R. Gegenfurtner

**Affiliations:** 1 Fachbereich Psychologie, Philipps-Universität Marburg, AG Sensomotorisches Lernen, Gutenbergstraße 18, 35039 Marburg, Germany; 2 Center for Mind, Brain and Behavior, Marburg, Gießen and Darmstadt, Germany; 3 Abteilung Allgemeine Psychologie, Justus-Liebig-Universität Gießen, Gießen, Germany

**Keywords:** Statistical inference, Confidence intervals, Repeated measures, Scatter plots

## Abstract

Scatter plots are a standard tool to illustrate the covariation of bivariate data. For paired observations of the same variable, they can also be used to illustrate differences in the central tendency. For these differences, it would be useful to draw confidence intervals (CIs) that correctly align with statistical analyses. Here, we describe a method to compute and draw a diagonal CI for pairwise differences in scatter plots. This CI can be compared to the identity line that marks coordinates with identical values in both observations. Such CIs offer advantages for both authors and readers: for authors, the CI is simple to compute and to draw; for readers, the CI is less ambiguous and more informative than other types of illustrations, because the three CIs of the standalone effects of *x, y* and their pairwise differences can be plotted simultaneously along horizontal, vertical and diagonal axes, respectively. A survey testing the interpretation of standalone effects and pairwise differences in bar and scatter plots by scientists showed that such effects can be interpreted with high certainty and accuracy from scatter plots containing horizonal and vertical CIs for standalone effects and diagonal CIs for pairwise differences.

## Introduction

Scatter plots are the most common way to illustrate bivariate data sets (Cleveland & McGill, 1984). The typical application is to illustrate the covariation of bivariate data in correlation or regression analysis (Correll & Heer, 2017; Goh et al., 2022). For that application, the only requirement is that there are (at least) two observations on the same cases, which can be plotted on the abscissa and the ordinate of the scatter plot. A typical example would be the covariation of human body size and weight. Another application is the comparison of paired observations with respect to their central tendency. This additionally requires that the paired observations are given in the same measurement units, because a direct comparison would otherwise be meaningless. In a scatter plot, the location of data points can then be compared to the identity line (line with an intercept of zero and a slope of one, also called the equality or unity line) that marks coordinates with identical values on the abscissa and the ordinate. A typical example would be the body weight before and after a diet.

**Fig. 1:**
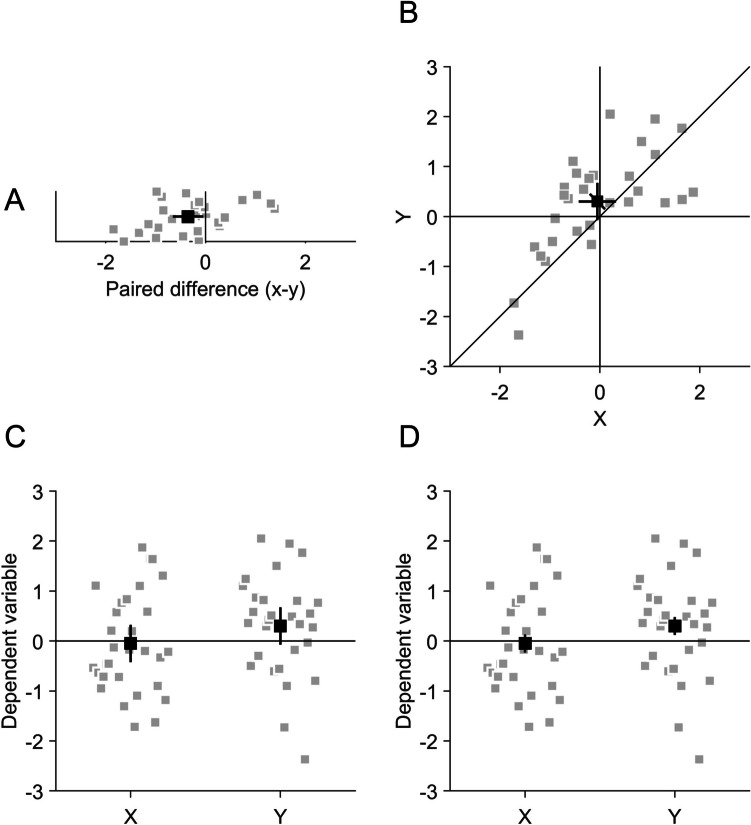
Different illustrations of a data set with paired observations. A sample of 30 was drawn from a bivariate standard normal distribution, with means of −0.05 and 0.3 for *x*- and *y*-values and a correlation of 0.6 between *x* and *y*. Grey data points indicate individual data, black data points indicate averages. **A** 1D representation of the pairwise differences between *x* and *y*. The black symbol indicates the mean of the pairwise differences and the error bar is the 95% confidence interval (CI) of the pairwise differences, which has to be compared to zero. Grey data points are jittered along the *y*-axis to facilitate recognition. **B** Scatter plot of *x*- and *y*-values. The diagonal error bar is the 95% within-subject CI of the pairwise differences, which has to be compared to the identity line that marks coordinates with identical values on the abscissa and the ordinate. Horizontal and vertical error bars are 95% between-subject CIs of the *x* and *y* standalone effects and have to be compared to the vertical and horizontal lines, respectively. **C** and **D** Bar-plot like representations of the data. Please note that it can be misleading to connect *x*- and *y*-values by lines when the abscissa contains a categorical variable because the slope of the connecting lines must not be interpreted. **C** Error bars are 95% between-subject CIs of the standalone effects of *x* and *y*. These error bars can be compared to the horizontal at zero to determine if *x*- and *y*-values independently are different from zero. The error bars are not informative about the pairwise differences between *x* and *y*, which depend on the correlation between *x* and *y*. **D** Error bars are 95% within-subject CIs of the pairwise differences of *x* and *y*, calculated according to Loftus and Masson (1994). The error bars are not informative about the difference of each, *x*- and *y*-values to zero

However, the widespread use of scatter plots for comparing the central tendency of paired observations is hampered by the fact that there is no widely known way of how to draw correct confidence intervals (CIs) for the within-subject comparison between paired observations in scatter plots. Previous publications on within-subject confidence intervals often neglected scatter plots and focused on bar or line graphs, in which all conditions are distributed along the ordinate and the abscissa shows the independent variable (Baguley, 2012; Cousineau, 2017; Cumming & Finch, 2005; Franz & Loftus, 2012; Loftus & Masson, 1994; Masson & Loftus, 2003; Pfister & Janczyk, 2013), as in Figs. 1C and D. Given that CIs are considered useful to illustrate statistical results (Masson & Loftus, 2003) and that their usage is recommend by academic societies, including the American Psychological Association (American Psychological Association, 2020) and the American Medical Association (Christiansen et al., 2020), and by scientific journals, including Nature (Nature Portfolio) and Science (American Association for the Advancement of Science), it seems to be particularly problematic that such CIs are not available for scatter plots.

**Fig. 2:**
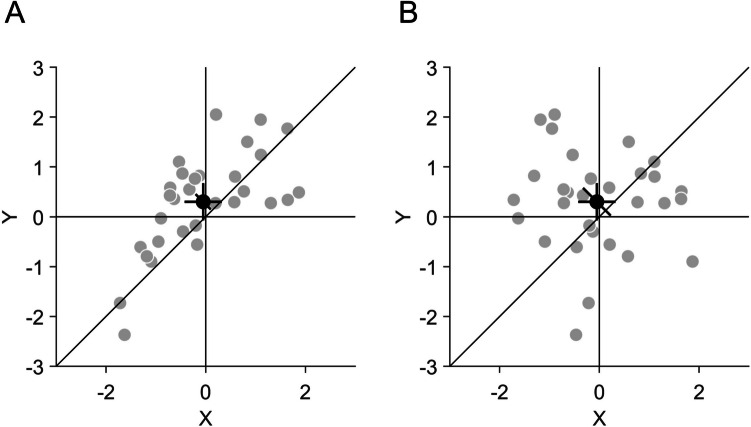
Illustration of the independence of confidence intervals (CIs) for standalone effects and pairwise differences. **A** The same data as in Fig. 1B are shown. *X*- and *y*-values are correlated (*r*(28) = 0.67, *p* < 0.001) and there is a significant difference between *x* and *y* (*t*(29) = −2.35, *p* = 0.026). **B** The same *x*- and *y*-values as in Fig. 2A are shown, but their correlation is broken by randomly permuting the *y*-values (*r*(28) = −0.10, *p* = 0.606). This leaves the standalone CIs for *x* and *y* unaffected, but increases the CI of the pairwise difference. There is no significant difference between *x* and *y* (*t*(29) = −1.29, *p* = 0.206)

The lack of a standard method to represent CIs for paired observations in scatter plots leads to a large variety of visualisations. A few arbitrarily selected examples from the fields of psychology and neuroscience are: only individual data points (e.g., Figure 6 in Mirpour et al., 2009; Figure 7 in Scheibehenne & Pachur, 2015); individual data points plus their mean without error bars (e.g., Figure 4 in Luu & Levi, 2013; Figure 6 in Mikellidou et al., 2018); individual data points plus their mean with horizontal and vertical error bars for the standalone effects of the two observations, in which the mean is either represented as one data point in two-dimensional coordinates (e.g., Figure 5 in Farell & Ng, 2019; Figure 2 in Hanning et al., 2024) or it is represented by two separate data points plotted on the abscissa and the ordinate (e.g., Figure 7 in García-Pérez & Peli, 2014; Figure 2 in Roberts & Carrasco, 2022); only the mean with standalone error bars (e.g., Figure 6 in Amadeo et al., 2019; Figure 2 in Veríssimo et al., 2024). In some cases, the scatter plot with individual data points is even supplemented by a bar graph to illustrate the means (e.g., Figure 2 in Abeles & Yuval-Greenberg, 2017; Figure 2 in Luo et al., 2024). All of those types of illustrations have in common that they lack a clear graphical representation of the CI of the paired comparison.

Here, we describe a simple procedure of how to draw diagonal within-subject CIs that can be compared to the identity line in a scatter plot. We present results of a survey with scientists showing that pairwise differences can be interpreted intuitively with high certainty and accuracy with such CIs.

## Construction of diagonal within-subjects confidence intervals (CIs) in scatter plots

### Methods

#### Calculation of the CI

The first step is to calculate a CI for the distribution of the differences between the paired observations, which can be compared to zero in a one-dimensional plot of the differences (Fig. 1A). Using the distribution of pairwise differences as the basis for within-subject CIs has been recommended as a parsimonious and bias-free method (Franz & Loftus, 2012; Pfister & Janczyk, 2013). Since we are dealing with the special case of comparing two paired observations in scatter plots, the calculation of the pairwise differences does not rest on any assumptions about variance homogeneity (for a discussion of that issue when comparing more than two measurements at once, see Cousineau, 2005; Franz & Loftus, 2012; Morey, 2008).

For the purpose of diagonal within-subject CIs in scatter plots, it does not matter how the CI of the pairwise differences is derived statistically (Cousineau, 2017; Morey et al., 2016), for example, if it is based on the standard error of the mean and Student’s t distribution (Student, 1908) in frequentist analyses, on Bayesian interval estimation (Kruschke, 2018) or on bootstrapping (DiCiccio & Efron, 1996). As an example, here are equations for the 95% CI based on the standard error of the mean of the pairwise differences and the t distribution. *X* and* y* denote the paired observations, *n* the number of pairs, and α the desired type-1 error:

1 $${SEM}_{pairedDiff}=\sqrt{\frac1{n\left(n-1\right)}\sum\nolimits_i^n\left(x_i-y_i-\left[\overline{x-y}\right]\right)^2}$$

2 $${CI}_{pairedDiff}={SEM}_{pairedDiff} {t}_{n-1,\alpha /2}$$

#### Drawing of the CI

In the second step, the obtained CI of the pairwise differences has to be transformed from a one-dimensional to a two-dimensional representation in the scatter plot. The CI could be directly plotted along the abscissa and the ordinate to extend from the mean $\left(\overline{x },\overline{y }\right)$ towards the two points of identity, $\left(\overline{x },\overline{x }\right)$ and $\left(\overline{y },\overline{y }\right)$, on the identity line because the distance between $\left(\overline{x },\overline{y }\right)$ and the points of identity is equal to the distance between zero and the mean of the pairwise differences. However, such horizontal or vertical CIs could be easily misinterpreted as the standalone CI of *x* or *y*. A more intuitive and elegant solution is to show the CI of the pairwise differences just once. To do so, the CI needs to be projected from the vertical (or the horizontal) axis between the mean $\left(\overline{x },\overline{y }\right)$ and the identity line to the perpendicular of the identity line to the mean. Since the vertical (or the horizontal) is the hypothenuse in a right triangle with the identity line and its perpendicular through the mean, we can use the Pythagorean theorem to project the CI to the perpendicular:

3 $${CI}_{pairedDiff\_Diagonal}= \frac{{CI}_{pairedDiff}}{\sqrt{2}}$$

To calculate the horizontal and vertical coordinates of the initial and end points of the diagonal CI, we need to apply the Pythagorean theorem again:

4 $${v}_{init}= \left(\overline{x }+{~}^{{CI}_{pairedDiff\_Diagonal}}\!\left/ \!{~}_{\sqrt{2}}\right.,\overline{y }-{~}^{{CI}_{pairedDiff\_Diagonal}}\!\left/ \!{~}_{\sqrt{2}}\right.\right)$$

5 $${v}_{end}= \left(\overline{x }-{~}^{{CI}_{pairedDiff\_Diagonal}}\!\left/ \!{~}_{\sqrt{2}}\right.,\overline{y }+{~}^{{CI}_{pairedDiff\_Diagonal}}\!\left/ \!{~}_{\sqrt{2}}\right.\right)$$

We can simplify Eqs. 3–5 by directly dividing the original CI of the pairwise differences by two to obtain the coordinates of the initial and end points:

6 $${v}_{init}= \left(\overline{x }+{~}^{{CI}_{pairedDiff}}\!\left/ \!{~}_{2}\right.,\overline{y }-{~}^{{CI}_{pairedDiff}}\!\left/ \!{~}_{2}\right.\right)$$

7 $${v}_{end}= \left(\overline{x }-{~}^{{CI}_{pairedDiff}}\!\left/ \!{~}_{2}\right.,\overline{y }+{~}^{{CI}_{pairedDiff}}\!\left/ \!{~}_{2}\right.\right)$$

#### Interpretation of the CI

The diagonal CI of the pairwise differences can fulfil multiple roles when interpreting data in a scatter plot. Note that the core of our proposal is to display the CI of pairwise differences along the perpendicular from the identity line to the mean data point. It is agnostic about the statistical derivation of the CI and its exact interpretation will depend on the chosen statistical approach. While CIs in general can allow for visual inferences (Coulson et al., 2010; Cumming & Finch, 2005), it also has been shown that their interpretation can be confusing even for experienced scientists (Belia et al., 2005; Cumming et al., 2004; Hoekstra et al., 2014). Therefore, it is important that authors clearly lay out for readers the underlying statistical approach and the resulting possibilities of interpretation of the CIs.

The CI can be used to supplement or replace classical tests for pairwise differences by comparing the CI to the identity line which marks locations with identical *x* and *y*-values (Fig. 1B). If the CI displays the 95% CI of the pairwise differences, it complies with the golden rule of CIs (Cousineau, 2017) in the sense that the paired observations can be considered as comparable when the CI crosses the identity line. For instance, the diagonal CI overlaps with the identity line in Fig. 2B, but not in Fig. 2A, which indicates that the *x*- and *y*-values are significantly different from each other in the latter but not in the former.

Beyond the mere binary testing for significant differences between the paired observations, the CI of the pairwise differences can also be used as a visual heuristic for other statistical estimates. The CI provides an illustration of the variance of the pairwise differences (given a fixed sample size), which corresponds to the sum of the variances of the standalone effects minus twice their covariance. A small CI illustrates that the variance of the pairwise differences is small or, in other words, that the covariance is large compared to the variances of the standalone effects. For example, in Fig. 2A the diagonal CI is quite small, indicating that although there is some variance of the standalone effects, their covariance is high. In contrast, in Fig. 2B, the CI of the pairwise differences is large, indicating that the covariance is small relative to the variance of the standalone effects. Illustrating the variance of the paired observations is particularly important because of two reasons: First, it cannot be estimated from the variances of the standalone effects alone and therefore cannot be inferred from the CIs of the standalone effects (Fig. 2). Second, illustrating the variance of the pairwise differences by the diagonal CI is particularly important if it cannot be illustrated by the individual data points. This could be the case if many data points are overlapping each other, such that the underlying distribution becomes obscured or if it is not possible to show the complete distribution because of extreme values. In these cases, plotting the mean with the diagonal CI of the pairwise differences can provide important information about the distribution of the data. Comparing the length of the CI to the distance between the mean and the identity line allows for a visual estimation of the effect size of the pairwise differences. Given a fixed sample size, the effect size increases with the distance to the identity line. This allows for a more fine-grained estimation of the effect beyond the binary classification of significance. Finally, the diagonal CI also gives an indication about the probabilities of the pairwise difference. Here, the exact interpretation of the CI depends on the underlying statistical procedure (Hoekstra et al., 2014). If the CI is calculated in a frequentist approach (for instance on the basis of a Student’s t-test as in Eq. 2), one could interpret that in 95% of cases, the CI would include the true mean difference between the paired observations. If a Bayesian credible interval is plotted (Kruschke, 2018; Morey et al., 2016), one could interpret that the mean difference between the paired observations lies with 95% probability within the CI.

### Recommendations for illustrating paired observations in scatter plots

In the following section, we discuss recommendations on how to illustrate paired observations in scatter plots: Some apply to illustrations of paired observations in scatter plots in general and some specifically to the diagonal CIs of the pairwise differences.

**Fig. 3:**
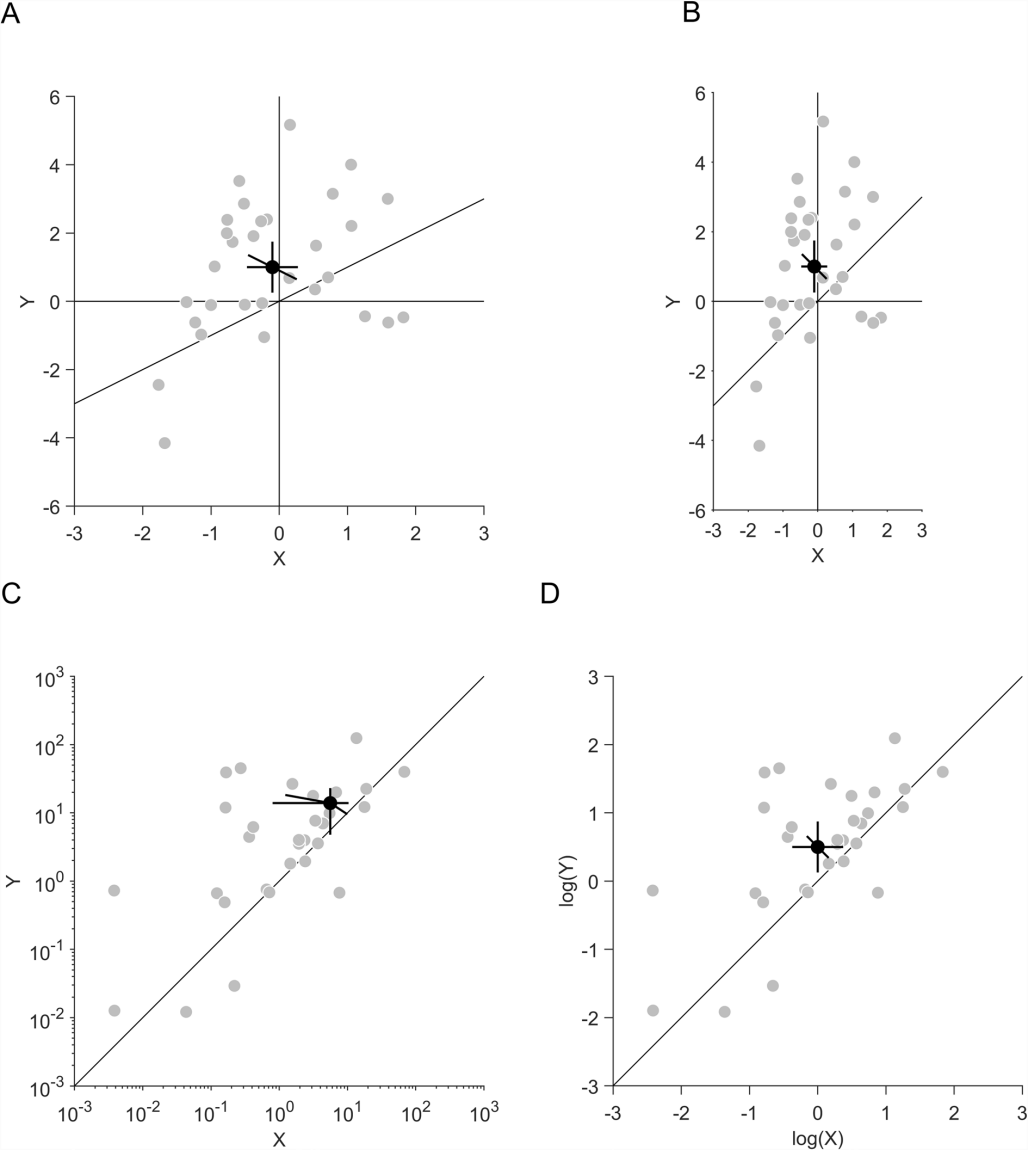
Illustration of paired observations in scatter plots. **A** and **B** Scaling of *x*- and *y*-axes. A sample of 30 was drawn from a bivariate normal distribution, with means of −0.1 and 1 for *x* and *y*, respectively; standard deviations of 1 and 2 for *x* and *y*, respectively, and a correlation of 0.3 between *x* and *y*. **A** Plot with magnified scale of the *x*-axis compared to the *y*-axis. **B** Plot with identical scales of *x*- and *y*-axes. **C** and **D** Log–log plots. A sample of 30 was drawn from a bivariate standard normal with means of 0 and 0.5 for *x* and *y*, respectively, and a correlation of 0.8 between *x* and *y*. These values were used as the exponent to a basis of 10. **C** Logarithmic axes scaling with confidence intervals (CIs) calculated in linear space. **D** Linear axes scaling, plotting log values and CIs calculated in log space. **A–D** Grey data points indicate individual data, black data points indicate averages. Horizontal, vertical and diagonal error bars indicate the 95% CIs of *x*, *y* and their pairwise differences, respectively

When displaying paired observations in scatter plots, it is advisable to use equal scaling of the horizontal and vertical axes (Figs. 3A and B) for three reasons: First, with equal scaling, unequal variances of *x* and *y* are directly visible from the scatter of individual data points and from the length of the horizontal and vertical CIs of the standalone effects. Scaling differences between the axes would dissociate visible from actual variances, making it harder to interpret the plot. Second, with equal scaling, the main diagonal has a slope of 1 (or an angle to the ordinate of 45°). Deviations from such angles are most easily discriminated (Cleveland et al., 1988). Third, it is not necessary to adjust the scaling of the axes to the variances in the standalone effects, because the correlation in a scatter plot can be estimated equally well whether the variances are equal or unequal (Rensink, 2017). There are additional suggestions on how to determine the aspect ratio of scatter plots (Doherty & Anderson, 2009; Fink et al., 2013).

Furthermore, it is advisable to calculate and display the CIs in the same space (Figs. 3C and D) to avoid potential spatial distortions. As an example, when confidence intervals are calculated with linear values but then displayed in logarithmic axes (Fig. 3C), horizontal and vertical CIs of the standalone effects can become asymmetric and diagonal CIs of the pairwise differences can become tilted. Hence, if the underlying distribution of the data is logarithmic, a better solution is to calculate the CIs of the log values and plot these values in a log–log plot (Fig. 3D). Similar distortions can occur when CIs are derived from logistic regression (with values ranging from negative to positive infinity) but plotted in proportion space (with values ranging from zero to one). This is a general problem that occurs whenever CIs are calculated in a different space to the one they are plotted in.

### Comparison with other types of illustrations of paired observations

As an alternative to a scatter plot, paired observations are often shown in a one-dimensional representation, where both dependent variables are distributed along the ordinate and the independent variable is plotted on the abscissa, such as in bar or violin plots (Figs. 1C and D). This is, of course, the best way to illustrate independent observations, but for paired observations, the scatter plot (Fig. 1B) has two advantages. First, it can convey more information, both with respect to the data pattern (Anscombe, 1973; Matejka & Fitzmaurice, 2017) and with respect to CIs. As mentioned above, scatter plots are ideal to illustrate the covariance of the paired observations (Ciccione & Dehaene, 2021; Rensink, 2017), which is not easily seen in other types of graphs like bar plots (especially if the abscissa is a categorical variable and data points from the paired observations must not be connected by lines). This covariation is not just relevant with respect to correlation or regression analysis but also useful to understand how reliable the differences in the means are. It has been recommended in neuroscience and psychology repeatedly to provide more information about the underlying distribution of the data in scientific graphs (Allen et al., 2012; Hehman & Xie, 2021; Lane & Sándor, 2009; Weissgerber et al., 2015), and scatter plots can fulfil that objective. Furthermore, a scatter plot can convey more information because it can contain multiple CIs at the same time. In addition to the diagonal CI for the paired differences, the scatter plot can contain horizontal and vertical CIs for the two observations separately, which can be useful when both values are to be compared to some external criterion. It is important to note that horizontal and vertical CIs are not informative about the paired differences (Fig. 2). Other plots typically contain only one type of CI and researchers have to decide whether to plot CIs for the observations separately or for their paired difference (Cousineau, 2017; Franz & Loftus, 2012; Loftus & Masson, 1994; Pfister & Janczyk, 2013).

A second advantage of the scatter plot is that the interpretation of the CIs is less ambiguous. As mentioned above, in a design with paired observations, two different types of CIs could be shown, either for the separate observations independently (Fig. 1C) or for their paired difference (Fig. 1D). This requires researchers to state in the figure legend which type of CI is shown and readers to understand if the CI is informative about the paired difference or about the observations independently. This distinction seems to be a difficult task even for experienced researchers (Belia et al., 2005). It has been suggested to plot two-tiered error bars, representing the variance both of the standalone effects as well as of the pairwise differences (Baguley, 2012). As these would be plotted along the same (vertical) axis, this still requires rather abstract reasoning of readers to understand which of the two parts of the error bar is informative about the standalone effects and about the pairwise differences. In a scatter plot, the two observations are distributed to separate axes, which allows to distinguish the different types of CIs by their orientation. Their orientation intuitively represents their association to the data. There are a several examples where such CIs have been used in scatter plots (e.g., Men et al., 2023; Schütz et al., 2007, 2008; Wolf & Lappe, 2020).

Of course, scatter plots are not suited for every purpose. They are typically limited to pairs of two observations. If more than two observations are present in the data, an obvious solution might be to plot different factor levels as multiple datasets in one scatter plot. However, in this case it is important to note that the diagonal CIs are only indicative of the comparison of the *x*- and *y*-values for a single comparison. Furthermore, the perception of correlation in one dataset might be impaired and biased by the other in the same scatter plot (Elliott & Rensink, 2015; Omae & Saiki, 2024). Alternatively, one could use a matrix of scatter plots with all possible combinations or one could draw the paired differences of all possible combinations on the ordinate (Franz & Loftus, 2012).

## Survey to evaluate the interpretability of confidence intervals in scatter plots

Since scatter plots with diagonal CIs have not been evaluated previously, we conducted a survey to test the certainty and accuracy with which standalone effects and pairwise differences can be interpreted in one-dimensional plots (such as classical bar graphs) and in two-dimensional scatter plots. Potential problems in the interpretation of CIs could be that CIs for standalone effects or pairwise differences are used to interpret non-matching effects, i.e., to infer pairwise differences based on CIs for the standalone effects or to infer standalone effects based on the CI of the pairwise differences (Belia et al., 2005). For two-dimensional scatter plots in particular, horizontal and vertical CIs that indicate the between-subject variance of the standalone effects might be erroneously compared to the main diagonal to infer the within-subject variance of the pairwise differences. To test implicitly which effects are interpreted based on the different CIs, we asked participants to indicate for the standalone effects and pairwise difference whether the effects are significant or not or if they are uncertain about it.

### Methods

#### Design and stimuli

**Fig. 4:**
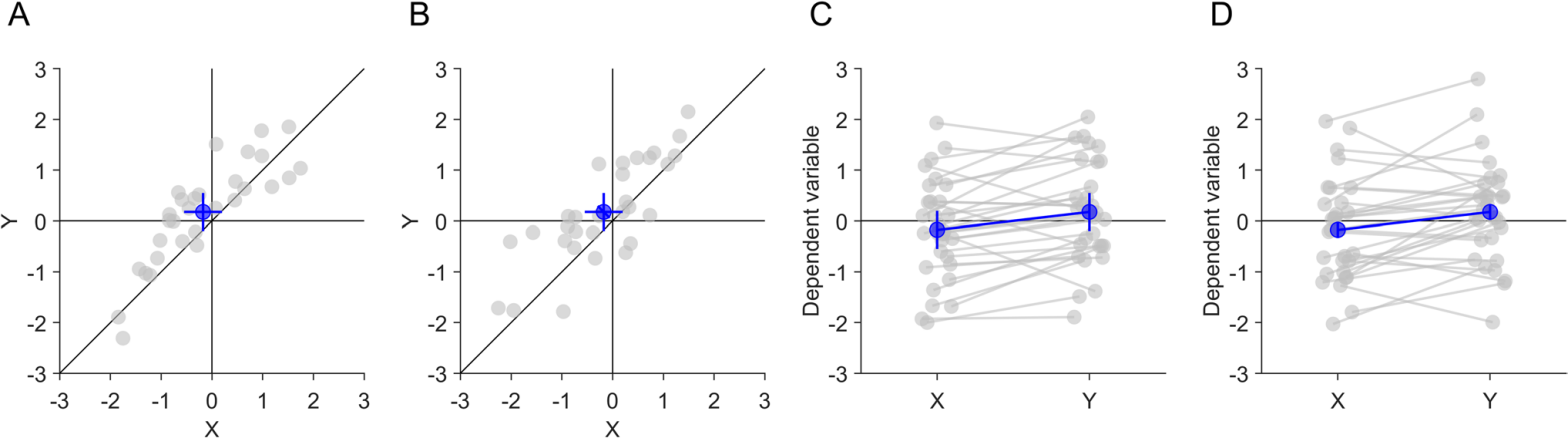
Compressed examples of the four display conditions. The data pattern #1 without significant standalone effects (*x*, *y*) but significant pairwise difference (*x*–*y*) is shown (Table 1). **A** 2D scatter plot with between-subject confidence intervals (CIs) for the standalone effects. **B** 2D scatter plot with between-subject CIs for the standalone effects and within-subject CI for the pairwise difference. **C** 1D plot with between-subject CIs for the standalone effects. **D** 1D plot with within-subject CIs for the pairwise difference

We used a two (plot layout: one-dimensional (1D) vs. two-dimensional (2D)) by two (CI: standalone vs. pairwise difference) by two (required inference: standalone effects vs. pairwise difference) design (Fig. 4) for the illustrations. One-dimensioanl plots were shown with either between-subjects CIs for the standalone effects or with within-subject CIs for the pairwise difference. Although there are methods to display both types of CIs in such plots (Baguley, 2012), we chose to show only one type of CI because we considered this to be the most frequently used version. Two-dimensional scatter plots were shown either only with conventional between-subject CIs for the standalone effects or with both between-subject CIs for the standalone effects and within-subject CIs for the pairwise differences. To make 1D and 2D plots more comparable, we showed individual data points and the average in both types of plots as dots. Paired observations were connected with lines in 1D plots to provide as much information as possible about their difference, apart from the CIs. These 1D plots provide much more information than classical and frequently used bar plots and therefore constitute a strong contender to the 2D scatter plot. Plots were displayed at a size of 500 × 500 pixels.

**Table 1:** Characteristics of data patterns

#	Significant effects	${x}$	${y}$	${x}-{y}$	${r}_{xy}$	${p}_{x}$	${p}_{y}$	${p}_{x}-{y}$
1	*x–y*	−0.175 [0.373]	0.175 [0.373]	−0.350 [0.204–0.271]	0.80	0.346	0.346	0.001—0.013
2	*x, y*	0.860 [0.373]	0.440 [0.373]	0.420 [0.515]	0.05	0.0001	0.023	0.106
3	*x*	0.500 [0.373]	0.250 [0.373]	0.250 [0.354–0.380]	0.51	0.010	0.181	0.159–0.188
4	*y, x–y*	−0.200 [0.373]	−0.500 [0.373]	0.300 [0.243–0.266]	0.75	0.282	0.010	0.018–0.029

The columns *x, y* and *x–y* provide the mean and the length of the 95% confidence interval (CI) of the standalone effects and the pairwise differences, respectively. The columns *p*_*x*_, *p*_*y*_ and *p*_*x-y*_ provide the *p*-values for the test of the standalone effects and the pairwise differences against zero, respectively

Each of the conditions was shown with four different data patterns of two paired observations (Table 1), leading to 16 trials overall. The data patterns were chosen to include various combinations of significant and non-significant standalone effects and pairwise differences, which pose particular challenges for (mis)interpretation of effects in 1D plots and 2D scatter plots. Data pattern #1 included only a significant pairwise difference between *x* and *y*. It posed the challenge for estimating the non-significant standalone effects in 1D plots that the within-subject CI of the pairwise difference did not include zero (Fig. 4D). Participants using within-subjects CIs of the pairwise differences to infer standalone effects would be misled to the wrong answer. In 2D scatter plots, the data pattern #1 posed the challenge that horizontal and vertical between-subject CIs of the standalone effects overlapped with the diagonal (Fig. 4A). Participants comparing between-subject CIs of the standalone effects to the main diagonal to infer the pairwise difference would be misled to the wrong answer. Data pattern #2 included only two significant standalone effects of *x* and *y*. For estimating the pairwise difference in 2D scatter plots, it posed the challenge that the horizontal and vertical between-subject CIs of the standalone effects did not overlap with the main diagonal. Data pattern #3 included only a significant standalone effect of *x*. It posed the challenge for estimating the non-significant standalone effect of *y* in 1D plots that the within-subject CI of the pairwise difference did not include zero. Data pattern #4 included only a significant standalone effect of *y* and a significant pairwise difference between *x* and *y*. It posed the challenge for estimating the non-significant standalone effect of *x* in 1D plots that the within-subject CI of the pairwise difference did not include zero.

*X* and *y* distributions were drawn from 2D random Gaussian distributions with a given mean and correlation and a standard deviation of 1. Samples were redrawn until the desired p-values were reached. To avoid recognition of encountered data patterns, each plot condition used a different data pattern, leading to small variations in the p-value in some cases.

#### Participants

Participants were recruited in the labs of the authors and through the mailing list of a collaborative, interdisciplinary research centre (https://www.sfb-perception.de) at the universities of Gießen and Marburg. The survey was started 165 times, but only 72 runs were finished and yielded complete datasets. The final sample included 72 participants (37 female, 35 male). Their average age was 33.15 years (*SD* 7.27, range 24–74 years). The highest academic degree was Master (28) or PhD (44). The average duration of research experience was 8.61 years (*SD* 7.14, range 1–50). Participants identified themselves with various academic backgrounds: Psychology (53), Neuroscience (6), Computer Science (4), Engineering (4), Linguistics (2), Sport Science (2), Physics (2), Biology (1). The survey was conducted in accordance with the Declaration of Helsinki (1964) and approved by the local ethics committee of the Psychology Department at Marburg University (proposal number 2020-43k). All participants provided written informed consent. All participants were naïve with respect to the aims of the survey.

#### Procedure

**Fig. 5:**
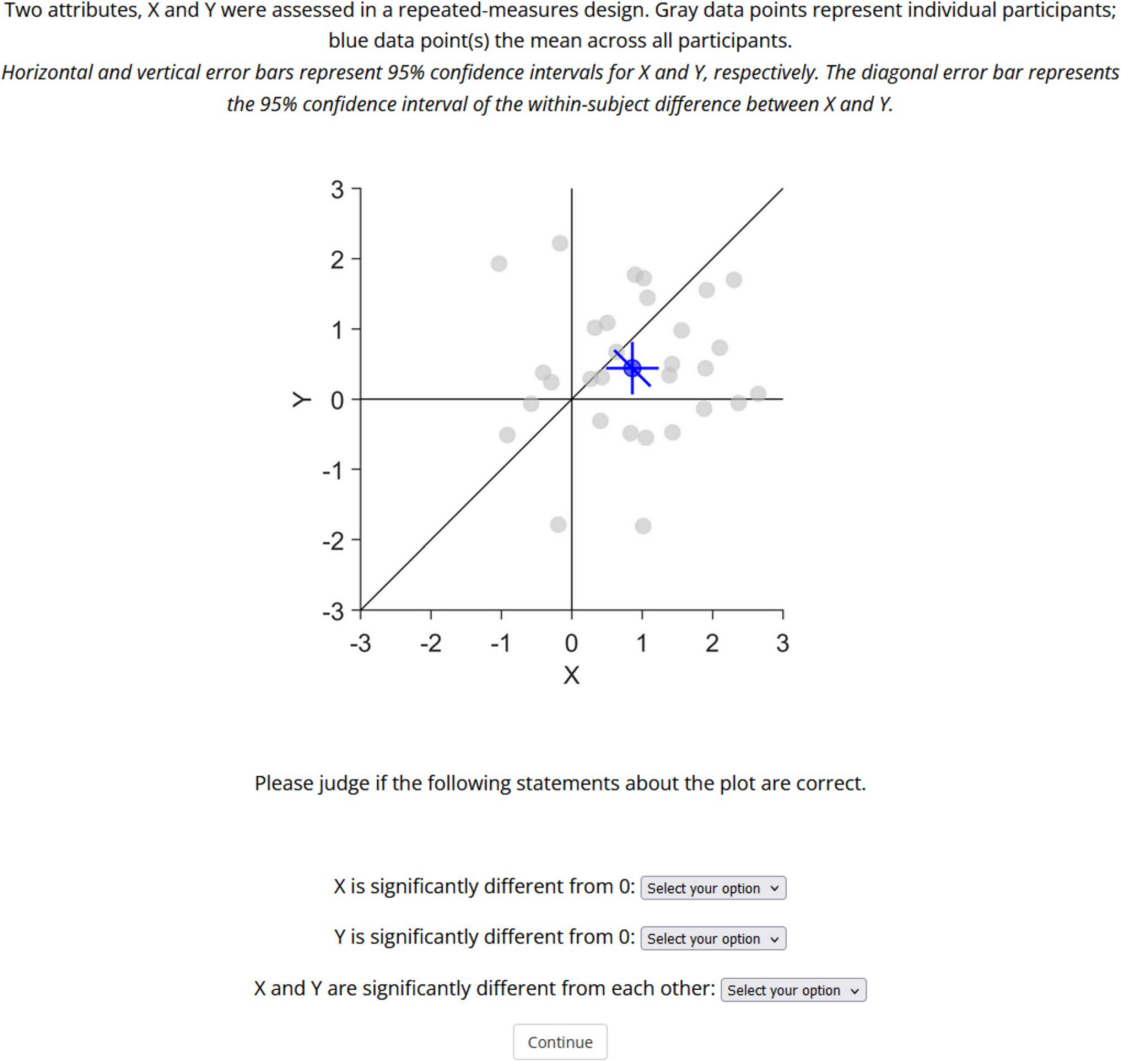
Example screen of one trial, showing a 2D scatter plot containing between-subject confidence intervals (CIs) for the standalone effects and within-subject CIs for the pairwise difference. The data pattern #2 contains significant standalone effects (*x, y*) but no significant pairwise difference (*x–y*). The first sentence was identical in all trials, the second sentence described the type of error bars in the figure below

Participants were initially asked to provide informed consent and to provide some demographic information (sex, age, academic background, highest academic degree and years of research experience). In each trial, participants saw a short vignette describing the plot and the meaning of the displayed errors bars (Fig. 5). Then they had to respond to three questions, whether individually *x* and *y* were significantly different from zero and whether *x* and *y* were significantly different from each other. Each question could be answered with yes, no or unknown. Participants had to answer all three questions before proceeding to the next trial. The order of trials was randomised for each participant. At the end of the experiment, they were asked if they encountered any technical issues or if they have any feedback. The survey was created with JsPsych (Leeuw et al., 2023).

#### Analysis

Based on the raw responses of the participants, we created two dichotomous response variables: response certainty that codes certain (yes or no) versus uncertain (unknown) responses and response correctness that codes if certain responses (yes vs. no) were correct or incorrect. Both response variables were analysed with a binomial general linear mixed effects model with plot layout, CI and effect type as fixed effects and participant as random effect with random intercept. Effect coding was used to test the influence of the fixed effects. Estimated marginal means and their CIs are reported.

### Results

**Fig. 6:**
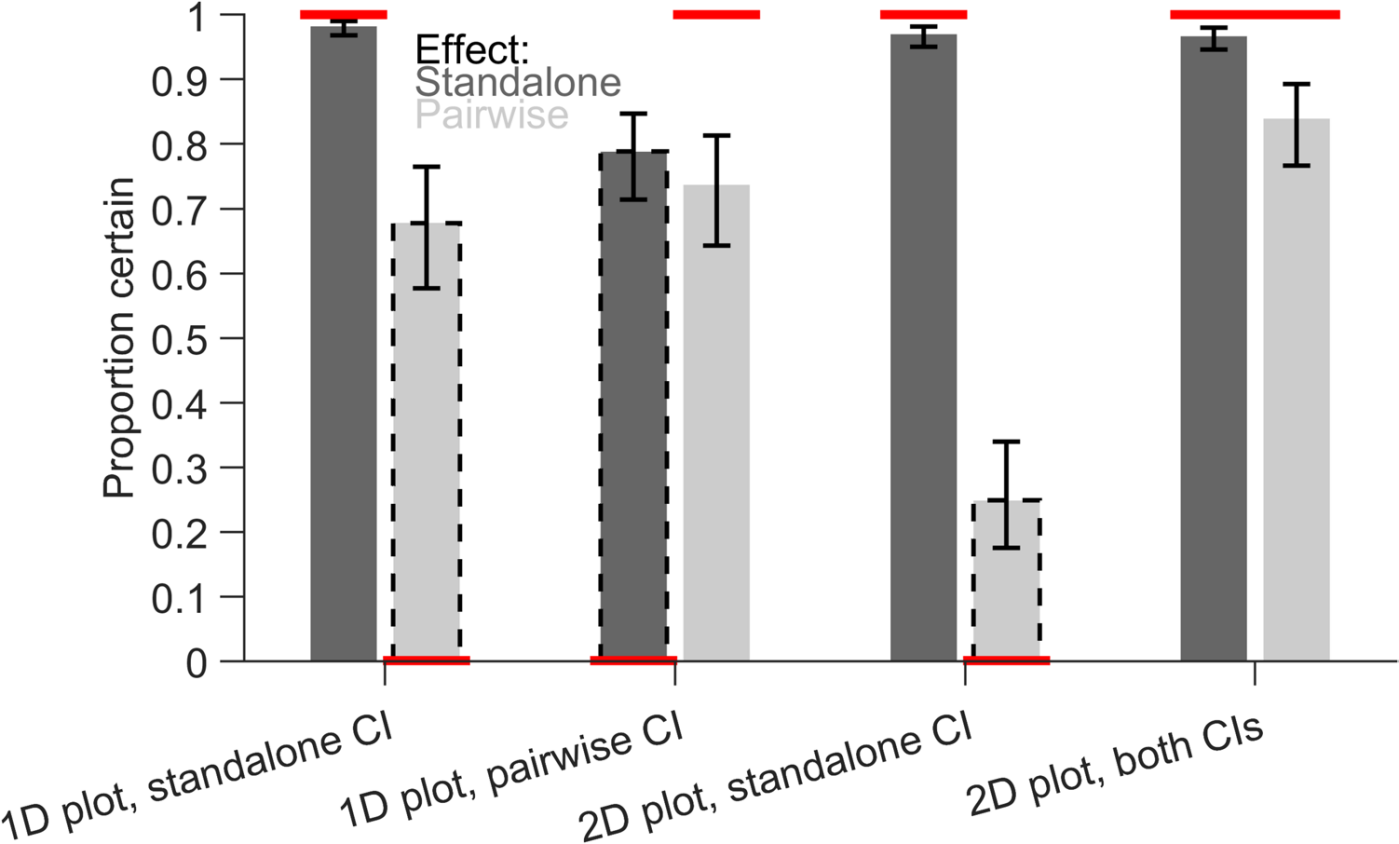
Proportion of certain responses. Estimated marginal means of the generalized linear model are shown with 95% confidence intervals (CIs). Dark and light bars indicate standalone effects and pairwise differences, respectively. Dashed outlines indicate non-matching combinations, where CIs in the plot are not informative about the effect. The horizontal red lines indicate expected values depending on the validity of the CIs

We first analysed the response certainty, i.e., how often participants chose a yes or no answer over the unknown answer (Fig. 6). We expected certainty to be high for matching CIs (i.e., standalone effects interpreted based on standalone CIs and pairwise differences interpreted based on within-subject CI of pairwise differences) and to be low for non-matching CIs (i.e., standalone effects interpreted based on within-subject CI of pairwise differences and pairwise difference interpreted based on standalone CIs). There were no main effects of plot layout (*F*(1,3448) = 0.39, *p* = 0.532) or CI (*F*(1,3448) = 0.28, *p* = 0.595) but a main effect of effect type (*F*(1,3448) = 377.42, *p* < 0.001). This main effect indicated that standalone effects were estimated with higher certainty than pairwise differences. All two-way interactions were significant: plot layout and CI (*F*(1,3448) = 109.68, *p* < 0.001), plot layout and effect type (*F*(1,3448) = 33.32, *p* < 0.001) and CI and effect type (*F*(1,3448) = 144.68, *p* < 0.001). The three-way interaction was not significant (*F*(1,3448) = 0.04, *p* = 0.830).

For standalone effects, the estimated marginal means showed high certainty (all values > 0.966) except for the 1D plot with within-subject CIs of pairwise differences (0.788, [0.714, 0.847]). Since the within-subject CIs of pairwise differences were not informative about the standalone effect in this case, participants were overconfident to judge these effects. For pairwise differences, the certainty was especially low for the 2D scatter plot with between-subject CIs of the standalone effects (0.249, [0.175, 0.340]), while there was only a small difference for 1D plots (between-subject CI of standalone effects: 0.678, [0.577, 0.765]; within-subject CI of pairwise differences: 0.737, [0.643 0.813]). Similar to the standalone effects, this suggests that participants were overconfident in the 1D plot about using non-matching CIs to interpret an effect (i.e., between-subject CIs of the standalone effects applied to the pairwise differences). Overall, the 2D scatter plot with between-subject CIs of the standalone effects and within-subject CIs of pairwise differences reached the highest levels of certainty.

**Fig. 7:**
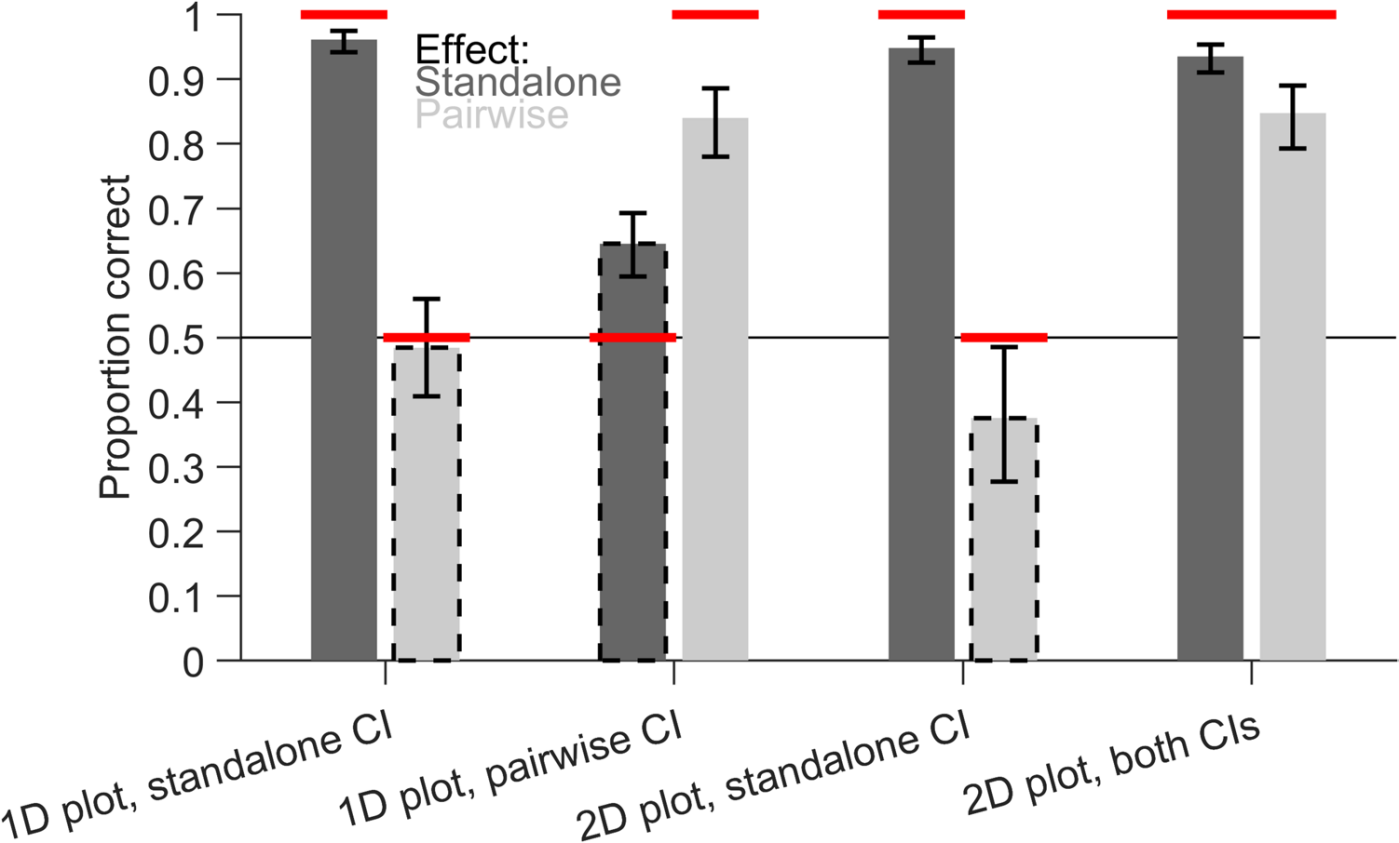
Proportion of correct responses (given a certain response). Estimated marginal means of the generalized linear model are shown with 95% confidence intervals (CIs). Dark and light bars indicate standalone effects and pairwise differences, respectively. Dashed outlines indicate non-matching combinations, where CIs in the plot are not informative about the effect. The horizontal red lines indicate expected values depending on the validity of the CIs

In the next step, we analysed response accuracy, i.e., how often yes or no responses corresponded to the actual effect in the data pattern (Fig. 7). We expected accuracy to be high for matching CIs and to be close to chance for non-matching CIs. All main effects, two-way interactions and the three-way interaction (*F*(1,2730) = 12.87, *p* < 0.001) were significant. For standalone effects, the estimated marginal means showed nearly perfect accuracy (all values > 0.934) except for the 1D plot with within-subject CIs of pairwise differences (0.645, [0.594, 0.693]). This is a similar data pattern to that for the response certainty and shows that the certainty to use non-matching CIs for interpretation was not warranted in this case because participants often gave the wrong answer. Nevertheless, they performed better than chance, indicating that they might have used the distribution of individual data points to estimate the standalone effects. For pairwise differences, the accuracy was low, close to chance for 1D plots (0.484, [0.409,0.560]) and even significantly lower than chance for 2D scatter plots (0.376, [0.277, 0.486]), with between-subject CIs of the standalone effects. The accuracy was high for 1D plots (0.840, [0.780, 0.886]) and 2D scatter plots (0.848, [0.793, 0.890]) containing within-subject CIs of pairwise differences. This shows that participants could not correctly interpret the pairwise differences based on the between-subject CIs of standalone effects in both 1D plots and 2D scatter plots. Overall, the 2D scatter plot with between-subject CIs of standalone effects and within-subject CIs of pairwise differences reached the highest levels of accuracy.

### Discussion

In a survey of scientists, we found that standalone effects and pairwise differences can be inferred with high certainty and accuracy in scatter plots containing both types of CIs. For standalone effects, certainty and accuracy were reduced for 1D plots with pairwise-differences CIs. This suggests that participants were to some extent aware that these CIs are not informative about the standalone effects. For pairwise differences, accuracy was reduced to chance level for 1D plots and below chance level for 2D scatter plots with standalone CIs. Importantly, in these cases, certainty was only reduced for 2D scatter plots, but not for 1D plots, indicating that participants felt overly confident to interpret pairwise differences in 1D plots with standalone CIs. The low certainty for 2D scatter plots in this condition also means that the below-chance accuracy should not be overinterpreted. In the majority of trials, participants were adequately stating that they cannot reach a certain decision based on the information in the figure, and as a result, there were only few responses left to analyse response accuracy regarding significance.

Although methods have been proposed to include both types of CIs in 1D plots (Baguley, 2012), we used 1D plots with one type of CI as benchmark. In our experience, this is still the most common option and therefore constitutes a realistic comparison. It might be that statistical inference in 1D plots could be improved by showing both types of CIs (Baguley, 2012).

The survey focused on simple decisions about the significance of standalone effects and pairwise differences because we were primarily interested if the association of horizontal and vertical CIs to standalone effects and the diagonal CI to the pairwise differences can be understood intuitively and how the different CIs are compared to the identity line. Obviously, CIs communicate more information about the distribution of a sample than the mere significance of an effect. However, it has been shown that there is quite some confusion about how to interpret CIs in general (Hoekstra et al., 2014). These problems, however, are not specific to the case of CIs for pairwise differences.

In sum, our results indicate that scatter plots containing CIs for standalone effects and pairwise differences can be interpreted intuitively with high accuracy and certainty. One-dimensional plots with either of these CIs are comparatively more difficult and more limited in their interpretation.

## General discussion

We described how CIs for pairwise differences can be computed and drawn in scatter plots of paired observations. A survey amongst scientists showed that both standalone effects and pairwise differences can be interpreted with high certainty and accuracy in such plots.

Drawing a diagonal CI for pairwise differences in scatter plots has multiple advantages. First and foremost, it leads to more accurate statistical inferences because, as our survey showed, it avoids the (common) misinterpretation to infer pairwise differences from CIs for the standalone effects (Belia et al., 2005). Second, it provides an illustration of the variance of the pairwise differences, which corresponds to the covariance of the paired observations relative to the variances of the standalone effects. Third, it also allows to visually estimate the effect size of the pairwise differences, given a certain sample size. This can facilitate the understanding of the relationship of the paired observations, especially in cases where the individual data points cannot be shown.

The proposed method to illustrate paired observations in scatter plots with separate CIs for the standalone effects and the pairwise difference can be supplemented by additional information. Similar to corset plots, the independent distributions of *x* and *y* values could be shown along the horizontal and vertical axis as histograms or smoothed density. This could be especially useful when there are too many observations to plot them as individual data points in the scatter plot. Since the two distributions would only illustrate the variability of the individual measurements, but not their covariance, it would be particularly important to display the diagonal CI of the pairwise differences in such a case. The proposed CI for the pairwise difference can also be applied to diamond plots, which are scatter plots rotated by 45° to avoid a causal interpretation between the values on the horizontal and vertical axis (Bergstrom & West, 2018).

Besides comparing the central tendency of paired observations, scatter plots are most frequently used to illustrate the covariation of bivariate data in regression analysis. In the special case of paired observations, where both *x*- and *y*-values are associated with some measurement error, traditional linear regression is not appropriate because it assumes that the *x*-values are noise-free and only considers vertical errors relative to the regression line. In the case of paired observations, Deming regression (Adcock, 1878; Deming, 1964; Kummell, 1879; Linnet, 1993) is more appropriate because it minimizes the Euclidean distance to the regression. Interestingly, human regression estimations are more consistent with Deming than with linear regression (Ciccione & Dehaene, 2021).

## Conclusions

Diagonal within-subject CIs of pairwise differences in scatter plots offer many advantages: they are simple to compute and to draw, they avoid some ambiguities that are involved in CIs in traditional one-dimensional plots, such as bar plots, they avoid confusions between CIs for standalone effects and pairwise differences and they illustrate the variance of the pairwise differences. Hence, such diagonal within-subject CIs of pairwise differences can be used to illustrate paired observations and should be implemented in statistical software packages.

## Data Availability

Data of the survey are available at 10.5281/zenodo.16275532.

## Code availability

Matlab, Python and R scripts for generating diagonal within-subject CIs in scatter plots are available at https://osf.io/2r4ux/. Data and scripts for reproducing the figures and analysis in the manuscript are available at 10.5281/zenodo.16275532.

## Declarations

### Open practices statement

Matlab, Python and R scripts for generating diagonal within-subject CIs in scatter plots are available at https://osf.io/2r4ux/. Data and scripts for reproducing the figures and analysis in the manuscript are available at 10.5281/zenodo.16275532. The survey was not preregistered.
